# Activation of mitophagy and proteasomal degradation confers resistance to developmental defects in postnatal skeletal muscle

**DOI:** 10.1186/s12929-025-01153-7

**Published:** 2025-08-19

**Authors:** Fasih A. Rahman, Mackenzie Q. Graham, Alex Truong, Joe Quadrilatero

**Affiliations:** Department of Kinesiology and Health Sciences, University of Waterloo, 200 University Ave. West, Waterloo, ON N2L 3G1 Canada

**Keywords:** Skeletal muscle, Mitochondria, Development, Mitophagy, Autophagy, UPS, Apoptosis, BNIP3

## Abstract

**Background:**

Postnatal skeletal muscle development leads to increased muscle mass, strength, and mitochondrial function, but the role of mitochondrial remodeling during this period is unclear. This study investigates mitochondrial remodeling during postnatal muscle development and examines how constitutive autophagy deficiency impacts these processes.

**Methods:**

We initially performed a broad RNA-Seq analysis using a publicly available GEO database of skeletal muscle from postnatal day 7 (P7) to postnatal day 112 (P112) to identify differentially expressed genes. This was followed by investigation of postnatal skeletal muscle development using the mitophagy report mouse line (mt-Kiema mice), as well as conditional skeletal muscle knockout (*Atg7*^f/f:*Acta1*−Cre^) mice.

**Results:**

Our study observed rapid growth of body and skeletal muscle mass, along with increased fiber cross-sectional area and grip strength. Mitochondrial maturation was indicated by enhanced maximal respiration, reduced electron leak, and elevated mitophagic flux, as well as increased mitochondrial localization of autophagy and mitophagy proteins. Anabolic signaling was also upregulated, coinciding with increased mitophagy and fusion signaling, and decreased biogenesis signaling. Despite the loss of mitophagic flux in skeletal muscle-specific *Atg7* knockout mice, there were no changes in body or skeletal muscle mass; however, hypertrophy was observed in type IIX fibers. This lack of *Atg7* and loss of mitophagy was associated with the activation of mitochondrial apoptotic signaling as well as ubiquitin–proteasome signaling, suggesting a shift in degradation mechanisms. Inhibition of the ubiquitin–proteasome system (UPS) in autophagy-deficient skeletal muscle led to significant atrophy, increased reactive oxygen species production, and mitochondrial apoptotic signaling.

**Conclusion:**

These results highlight the role of mitophagy in postnatal skeletal muscle development and suggest that autophagy-deficiency triggers compensatory degradative pathways (i.e., UPS) to prevent mitochondrial apoptotic signaling and thus preserve skeletal muscle integrity in developing mice.

## Introduction

During skeletal muscle embryonic development, the dermomyotome gives rise to PAX3^+^/PAX7^+^ muscle progenitor cells that proliferate and activate into committed myogenic cells, called myoblasts [1]. Following birth, skeletal muscle continues to grow (i.e., hypertrophy) and is accompanied by the proliferative activity of myogenic cells [2]. These cells donate their nuclei to the growing fibers to increase the overall number of myonuclei and expand their myonuclear domain [3, 4]. In mice, the first three weeks of postnatal development is accompanied by rapid increase in body weight up to eightfold, half of which is due to the increase in muscle mass [5]. Unsurprisingly, postnatal development of skeletal muscle is also accompanied by a robust increase in mitochondrial protein content [6], signifying an increase in energy demand. Importantly, there is a change in mitochondrial morphology during postnatal development. For example, the mitochondrial network adapts from a parallel to a perpendicular morphology relative to the fiber orientation [6, 7].

The development of the mitochondrial network may be attributed to the balance of mitochondrial remodeling processes, including mitochondrial dynamics and turnover. Mitochondrial dynamics involves the continuous cycles of fusion and fission, which regulate mitochondrial shape, distribution, and function [8]. Fusion helps maintain mitochondrial integrity by mixing contents, while fission facilitates the removal of damaged mitochondria and supports organelle distribution [8]. The importance of these processes has been signified during skeletal muscle development using experimental models. For instance, skeletal muscle-specific knockout of *Dnm1l*, *Opa1*, and *Mfn1/2*, proteins involved in mitochondrial fission and fusion, dramatically reduces lifespan and is associated with defects in skeletal muscle growth and mitochondrial dysfunction [9–11]. Although there is a clear change in mitochondrial morphology during postnatal development, the same is not entirely understood with respect to mitochondrial turnover (i.e., mitophagy and biogenesis). In particular, mitophagy (a specialized form of autophagy) plays a crucial role in maintaining mitochondrial quality by selectively degrading damaged or dysfunctional mitochondria [8]. This process utilizes the autophagic machinery (e.g., autophagosome), including specialized receptors and other sensors, which recognize and target mitochondria for degradation, to maintain cellular health.

Previous work from our lab has demonstrated that constitutive knockout of *Atg7*, an important autophagy protein, can result in smaller and weaker skeletal muscles that exhibit upregulated mitochondrial apoptotic signaling and proteasomal activity [12]. Additionally, there is emerging evidence suggesting the role of mitophagy as an initiator of mitochondrial biogenesis in myoblasts [13, 14], the lack of which can lead to increased mitochondrial apoptotic signaling including greater activation of CASP3, CASP9, and CAPN activity [13, 15]. Nonetheless, more work is needed to fully understand this mechanism with respect to postnatal skeletal muscle development. The purpose of this study was to investigate the role of mitochondrial remodeling during postnatal skeletal muscle development. The current study also explored the role of constitutive autophagy-deficiency and its implication on mitochondrial remodeling processes during postnatal muscle development.

## Results

### Postnatal skeletal muscle development exhibits significant changes in the genetic signature related to mitochondria

**Fig. 1:**
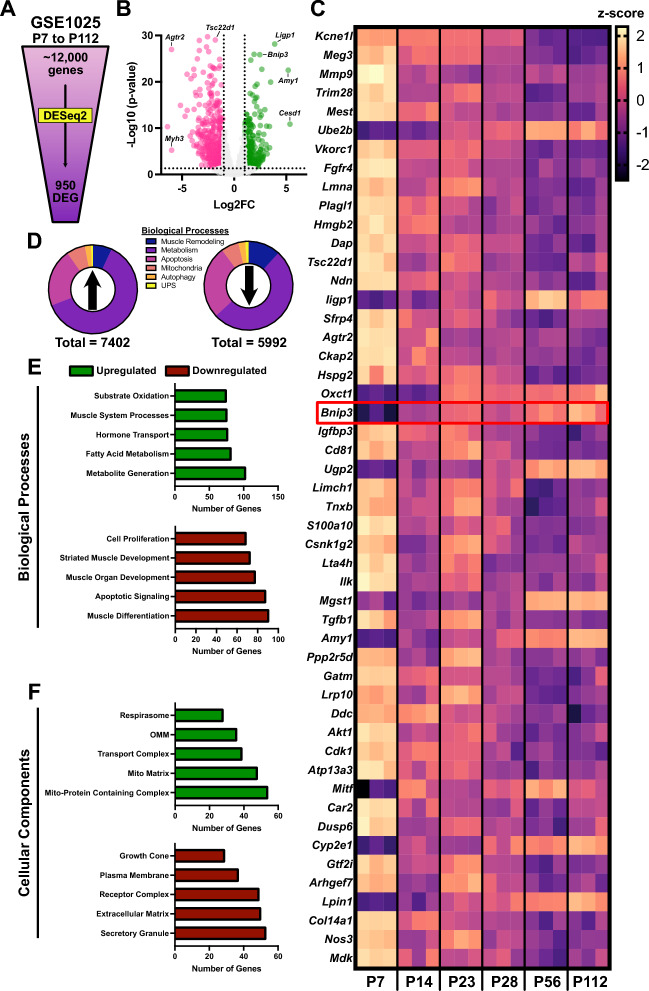
RNA sequencing analysis of gastrocnemius-plantaris muscle during development. **A** Representative visualization of genes analyzed from the publicly available Gene Expression Omnibus database (Accession ID: 1025). **B** Volcano plot displaying differential gene expression in skeletal muscle. The horizontal line delineates a significance threshold of p < 0.05. Vertical lines mark a z-score change greater than Log2. Green labels indicate upregulated differentially expressed genes (DEGs), while red labels indicate downregulated DEGs. **C** List of top 50 DEGs identified by DESeq2 analysis. **D** Gene ontology analyses and donut graph outputs summarizing changes in genes associated with five major remodeling processes: muscle remodeling, metabolism, apoptosis, mitochondria, autophagy, and the ubiquitin proteasome system (UPS). A total of 7402 genes related to these processes are upregulated while 5992 genes related to these processes are downregulated. **E, F** Gene ontology analyses output of the number of genes linked to the top five upregulated (green) and downregulated (red) biological processes. P7-P112 refers to animals collected at their respective postnatal timepoints. *N* = 3 mice per group

Postnatal skeletal muscle growth and development is a complex physiological process characterized by both hypertrophy of existing fibers and the remodeling of skeletal muscle architecture. Due to the limited number of studies on postnatal skeletal muscle development, we aimed to adopt a comprehensive approach to investigate this process. We performed RNA sequencing (RNAseq) analyses on developing skeletal muscle from the Gene Expression Omnibus (GEO) database (GSE1025). These data were collected from the gastrocnemius-soleus complex of young mice ranging from postnatal day 7 (P7) to postnatal day 112 (P112). Our RNAseq analysis of approximately 12,000 genes identified 950 differentially expressed genes (DEGs) during this developmental period. Among the DEGs, we found several genes related to extracellular remodeling, muscle growth, metabolism, and overall cellular signaling (Fig. 1A–C). It is interesting to note that among the top 950 DEG, only two of them are directly related to remodeling processes of autophagy and mitophagy, namely *Atg7* (rank 222/950) and *Bnip3* (rank 21/950; highlighted in Fig. 1C), respectively. It is interesting to note that *Atg7* expression declined approximately fivefold during postnatal muscle development between P7 and P112, whereas *Bnip3* expression increased approximately eightfold.

Further analysis of the RNAseq dataset using Gene Ontology (GO) analyses revealed 7402 upregulated genes and 5992 downregulated genes (Fig. 1D). These genes are related to key biological processes involved in growth-related remodeling (i.e., muscle-specific, metabolism, apoptosis, mitochondria-related, autophagy, and UPS). Specifically, we observed the upregulation of genes associated with biological processes related to metabolism and mitochondrial components during development (Fig. 1E, F). Conversely, there was a downregulation of genes involved in de novo muscle formation, cell growth, and other cellular remodeling components (Fig. 1E, F).

### Postnatal skeletal muscle development is accompanied by greater mitophagic flux and enhanced mitochondrial function

**Fig. 2:**
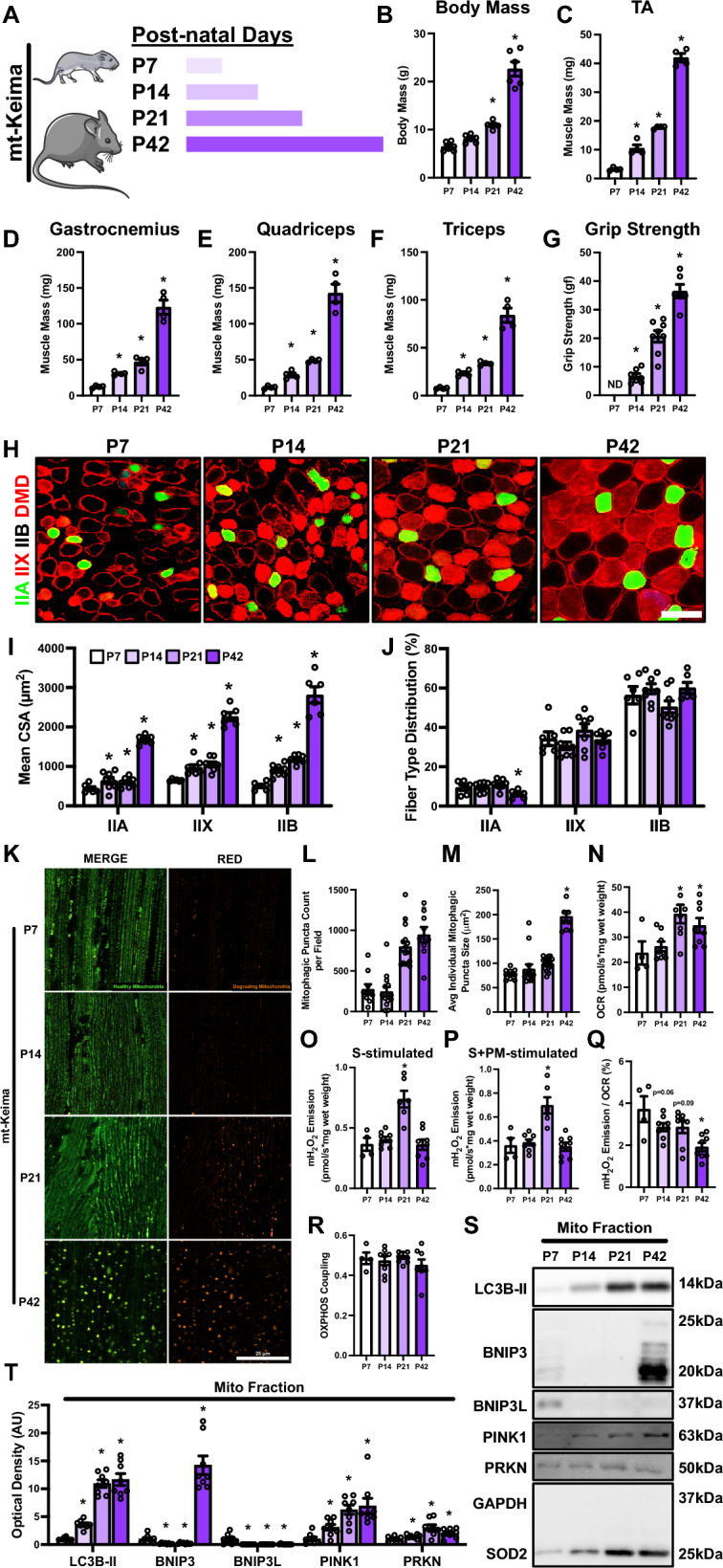
Changes in skeletal muscle during postnatal development of mt-Keima mice. **A** Representative visualization of collection timepoints. Quantification of **B** Body mass, relative skeletal muscle mass of **C** tibialis anterior (TA), **D** gastrocnemius, **E** quadriceps, and **F** triceps, and **G** peak forelimb grip strength. **H** Representative immunofluorescent images of TA muscle cross sections from P7 to P42 mice. Samples were stained with antibodies specific for individual MYH isoforms: type IIA (green), type IIX (red), type IIB (unstained), and dystrophin (DMD; red). Scale bars indicate 50 μm. **I**, **J** Quantification of mean cross-sectional area (CSA) and distribution of type IIA, IIX, and IIB fibers. **K** Representative confocal images of mitophagic flux from longitudinal TA muscle sections of mt-Keima reporter mice. Green fluorescence indicates healthy mitochondria. Red fluorescence indicates degrading mitochondria. Scale bar indicates 25 μm. **L** Quantification of mitophagic (i.e., red) puncta count per field. **M** Quantification of average individual mitophagic puncta size. **N** Quantification of maximal oxygen consumption rate (i.e., respiration) of permeabilized bundles from TA muscle. Simultaneous quantification of **O** succinate-stimulated hydrogen peroxide (H_2_O_2_) production, and **P** succinate + pyruvate/malate-stimulated H_2_O_2_ production in permeabilized TA muscle bundles. **Q** Quantification of mitochondrial fraction of electron leak. **R** Quantification of oxidative phosphorylation (OXPHOS) coupling. **S** Representative immunoblots of mitochondrial fractions derived from gastrocnemius. **T** Quantification of LC3B-II, BNIP3, BNIP3L, PINK1, and PRKN from mitochondrial-enriched fractions. GAPDH and SOD2 shown as subcellular fraction controls. * p < 0.05 compared to P7 group. *N* = 4–8 mice per group

To further investigate these physiological changes, we collected skeletal muscles from neonatal pure-bred mt-Keima reporter mice from P7 to P42 to understand the skeletal muscle growth pattern (Fig. 2A). We observed a steady rate of growth with respect to body mass and skeletal muscle mass from P7 to P21 (Fig. 2B–F). Importantly, we found a twofold increase in body mass and skeletal muscle mass from P21 to P42 (Fig. 2B–F). This increased skeletal muscle mass and cross-sectional area (CSA) were also accompanied by enhanced grip strength during development (Fig. 2G). Closer analyses revealed significant fiber hypertrophy within the tibialis anterior (TA), as shown by increased mean CSA of type IIA, IIX and IIB fibers. Once again, the most significant change observed was approximately a twofold increase in fiber CSA between P21 and P42 (Fig. 2H, I). Fiber type distribution remained identical across IIX and IIB fibers; however, relative proportion of IIA fibers decreased at P42 compared to P7 in TA muscle (Fig. 2J).

In line with the *Bnip3* DEG finding noted above, we observed greater mitophagic flux throughout the developmental time course (Fig. 2K). Specifically, we found increased mitophagic (i.e., red) puncta count at P21 that remained elevated at P42 (Fig. 2L), while the average red puncta size only increased at P42 compared to P7 (Fig. 2M). Interestingly, we did not observe a dramatic change in mitochondrial network morphology, which remained in a “parallel” configuration. To gain deeper insight into mitochondrial function, we conducted high-resolution respirometry coupled with simultaneous fluorometric detection of ROS. We detected greater maximal oxygen consumption rate (OCR) at P21 and P42 compared to P7 (Fig. 2N). Interestingly, succinate-stimulated (S-stimulated) and succinate plus pyruvate/malate-stimulated (S + PM-stimulated) mH_2_O_2_ emission was only elevated at P21 (~ twofold) compared to P7, and returned to similar levels by P42 (Fig. 2O, P). The fraction of electron leak steadily declined and was significantly depressed at P42 compared to P7 (-50%; Fig. 2Q), with no change in mitochondrial coupling (Fig. 2R). To further dissect the mechanism of mitophagy in developing muscles, we collected mitochondrial-enriched fractions and probed for select autophagy and mitophagy markers. We found mitochondrial localization of LC3B-II increased alongside BNIP3, PINK1, and PRKN during the developmental period (Fig. 2S, T). Interestingly, mitochondrial BNIP3L declined rapidly after P7 and was virtually undetectable from P14 onwards (Fig. 2S, T).

### Changes in mitochondrial remodeling complement postnatal skeletal muscle development

**Fig. 3:**
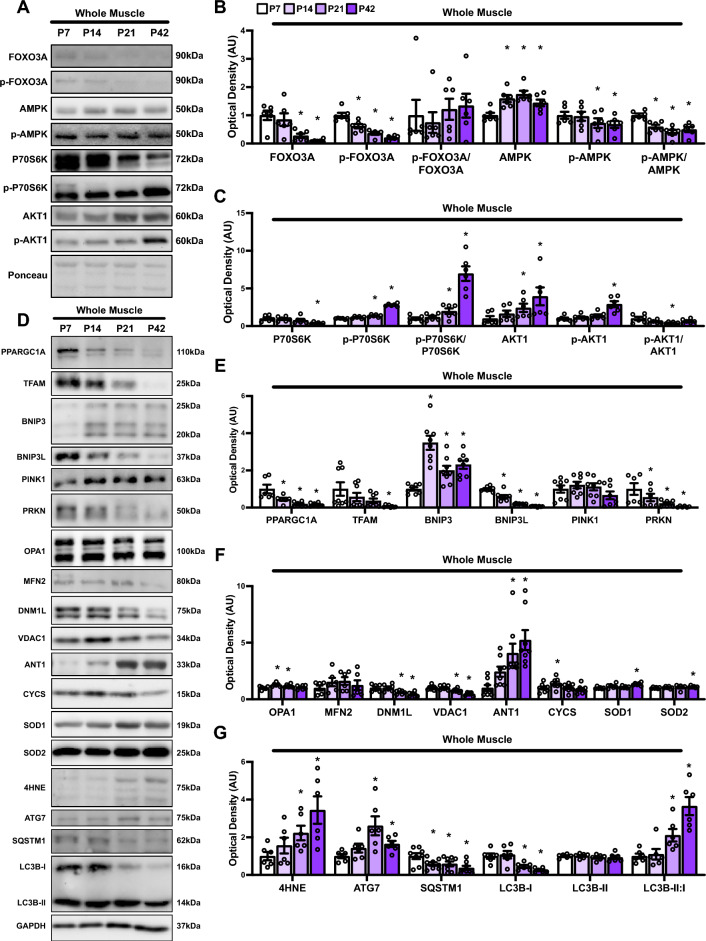
Quantitative analysis of upstream kinases and mitochondrial remodeling proteins in skeletal muscle during development. **A** Representative immunoblots of whole gastrocnemius lysate of mt-Keima mice. Ponceau stained membrane shown as loading control. **B** Quantitative analysis of FOXO3A, p-FOXO3A (Ser318/321), p-FOXO3A/FOXO3A, AMPK, p-AMPK (Thr172), and p-AMPK/AMPK. **C** Quantification of P70S6K, p-P70S6K (Thr421/424), p-P70S6K/P70S6K, AKT1, p-AKT1 (Thr308), and p-AKT1/AKT1. **D** Representative immunoblots of whole gastrocnemius lysate of mt-Keima mice. GAPDH shown as loading control. **E** Quantitative analysis of PPARGC1A, TFAM, BNIP3, BNIP3L, PINK1, and PRKN. **F** Quantification of OPA1, MFN2, DNM1L, VDAC1, ANT1, CYCS, SOD1, and SOD2. **G** Quantification of 4HNE, ATG7, SQSTM1, LC3B-I, LC3B-II, and LC3B-II:I. * p < 0.05 compared to P7. *N* = 6 mice per group

As noted in the results above, we observed a direct link between skeletal muscle growth and mitophagic flux. Although these signals are intuitively opposed, we questioned whether there were changes in upstream signaling kinases that regulate skeletal muscle growth (i.e., anabolic) and degradative (i.e., catabolic) pathways. FOXO3A and p-FOXO3A (Ser318/321) exhibited a gradual decrease from P7 to P42, leading to no significant change in the p-FOXO3A/FOXO3A ratio in mt-Keima mice (Fig. 3A, B). In contrast, total AMPK showed a notable increase throughout the developmental period from P7 to P42, accompanied by a decrease in p-AMPK (Thr172) at P21 and P42, resulting in an overall reduction in p-AMPK/AMPK ratio (Fig. 3A, B). While P70S6K was lower at P42, p-P70S6K (Thr421/424) was elevated at P21 and P42, resulting in higher p-P70S6K/P70S6K ratio at these time points in mt-Keima mice (Fig. 3A, C). Interestingly, we observed a consistent increase in AKT1 and p-AKT1 (Thr308), particularly at P21 and P42; however, this resulted in a decreased ratio (Fig. 3A, C). These data suggest that the ability of skeletal muscle to balance growth and degradation processes may involve fine-tuning of signaling pathways beyond mere upregulation of anabolic signaling.

Next, we measured protein levels of major molecules related to mitochondrial remodeling. We found a steady decline in markers of mitochondrial biogenesis (PPARGC1A and TFAM; Fig. 3D, E) and select mitophagy-related molecules (BNIP3L and PRKN; Fig. 3E) during postnatal skeletal muscle development from P7 to P42. Interestingly, we observed significant elevations in the mitophagy receptor BNIP3 (> twofold) and no changes in PINK1 (Fig. 3D, E). Changes in mitochondrial remodeling also include changes in mitochondrial dynamics. OPA1 was elevated at P14 and P21 compared to P7 before returning to baseline levels by P42 (Fig. 3D, F). In contrast, DNM1L significantly decreased at P21 and remained depressed at P42 compared to P7 (Fig. 3D, F), suggesting potential changes in mitochondrial dynamics that favor mitochondrial fusion. Furthermore, we found reductions in the OMM molecule VDAC1, particularly at P21 and P42, whereas the IMM molecule ANT1 significantly increased from P14 to P42 (Fig. 3D, F). Additionally, an elevation in CYCS was observed at P14, which returned to P7 levels by P21 (Fig. 3D, F). We also observed mild yet significantly elevated levels of SOD1 and SOD2 at P42 (Fig. 3D, F), along with a steady increase in the lipid peroxidation molecule 4-hydroxynonenal (4HNE; Fig. 3D, G). Autophagy molecules SQSTM1 and LC3B-I declined steadily throughout postnatal development, whereas ATG7 levels peaked at P21 and remained elevated at P42 (Fig. 3D, G). Additionally, the LC3B-II:I ratio increased dramatically over the same period (Fig. 3D, G). Together, these changes are indicative of increased autophagic activation. These results suggest a complex interplay between growth processes and mitochondrial dynamics, potentially mediated by nuanced alterations in both mitophagic and biogenesis pathways.

### Autophagy-deficiency abolishes mitophagic flux, modifies mitochondrial remodeling signals, yet does not impact overall skeletal muscle development

**Fig. 4:**
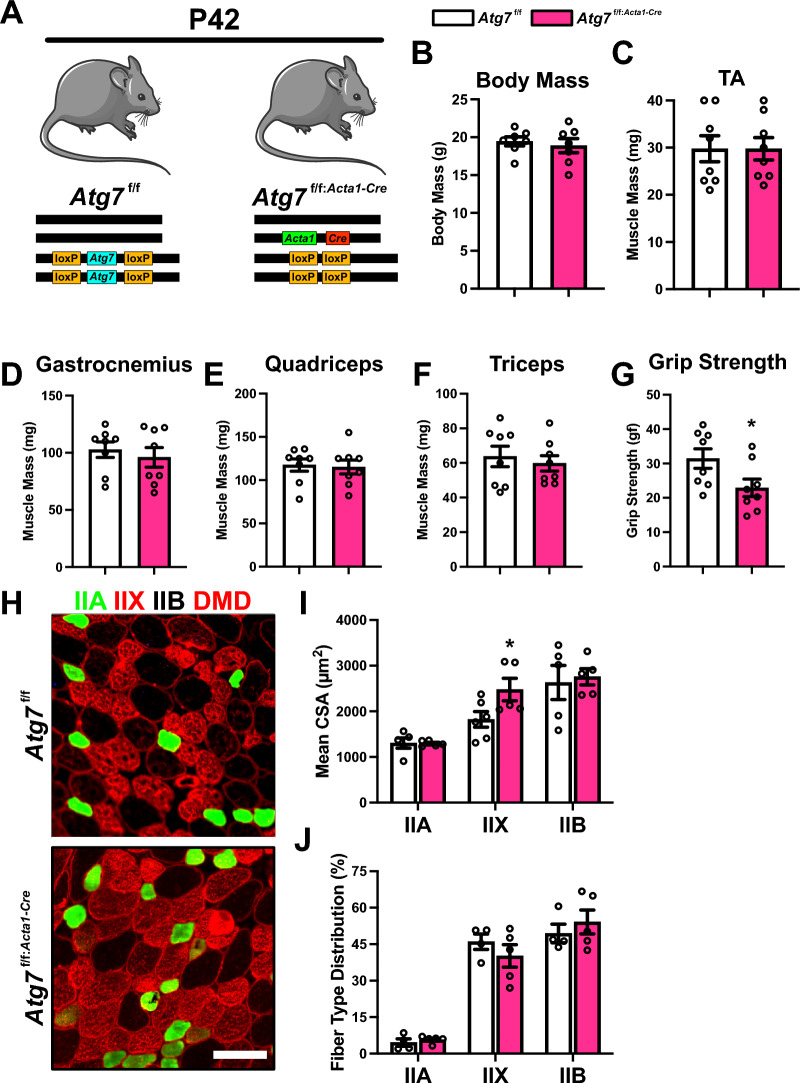
Developmental changes in skeletal muscles from *Atg7*^f/f^ and *Atg7*^f/f:*Acta1*−Cre^ mice. **A** Representative visualization of genotypes collected at P42. Quantification of **B** Body mass, relative skeletal muscle mass of **C** tibialis anterior (TA), **D** gastrocnemius, **E** quadriceps, and **F** triceps, and **G** peak forelimb grip strength. **H** Representative immunofluorescent images of TA muscle cross sections from P42 *Atg7*^f/f^ and *Atg7*^f/f:*Acta1*−Cre^ mice. Samples were stained with antibodies specific for individual MYH isoforms: type IIA (green), type IIX (red), type IIB (unstained), and dystrophin (DMD; red). Scale bars indicate 50 μm. **I**, **J** Quantification of mean cross-sectional area (CSA) and distribution of type IIA, IIX, and IIB fibers. * p < 0.05 compared to *Atg7*^f/f^ group. *N* = 4–8 mice per group

Recognizing that mitophagy can occur through various mechanisms, we broadened our approach to study changes in autophagy-deficient mice. We questioned whether autophagy-deficiency would negatively affect skeletal muscle growth by altering mitochondrial remodeling processes. In doing so, we generated a constitutive skeletal muscle-specific knockout mice by crossing *Atg7*^f/f^ mice with ACTA1-Cre mice, while *Atg7*^f/f^ mice were also used as controls (Fig. 4A). Despite no differences in body mass or skeletal muscle mass (i.e., TA, gastrocnemius, quadriceps, or triceps) between P42 *Atg7*^f/f^ and *Atg7*^f/f:*Acta1*−Cre^ mice (Fig. 4B–F), we observed a reduction in grip strength in *Atg7*^f/f:*Acta1*−Cre^ animals (Fig. 4G), consistent with our previous findings [12]. Fiber type analyses revealed hypertrophy in type IIX fiber CSA without changes in type IIA or IIB fiber CSA, as well as no shift in fiber type distribution (Fig. 4H–J).

**Fig. 5:**
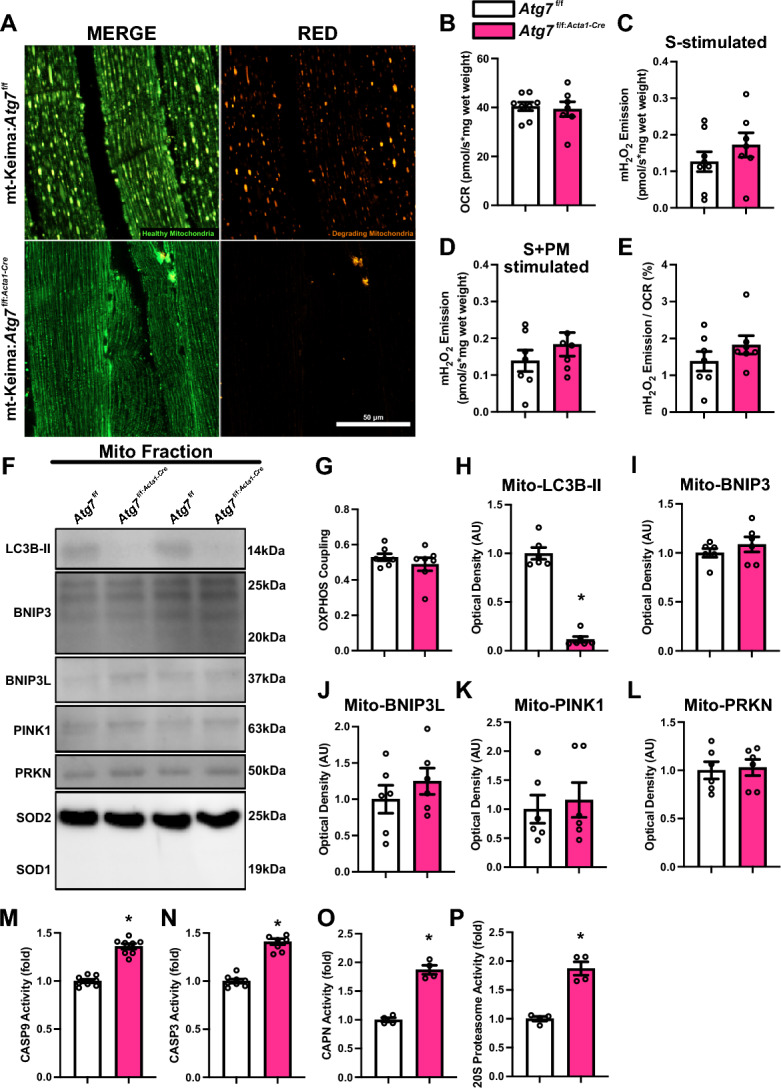
Postnatal development-associated changes in mitophagy and mitochondrial function in autophagy-deficient skeletal muscle. **A** Representative confocal images of mitophagic flux from longitutudinal TA muscle sections of *Atg7*^f/f^ and *Atg7*^f/f:*Acta1*−Cre^ mice crossed with mt-Keima reporter mice (mt-Keima:*Atg7*^f/f^ and mt-Keima:*Atg7*^f/f:*Acta1*−Cre^). Green fluorescence indicates healthy mitochondria. Red fluorescence indicates degrading mitochondria. Scale bar indicates 50 μm. **B** Quantification of maximal oxygen consumption rate (i.e., respiration) of permeabilized bundles from TA muscle. Simultaneous quantification of **C** succinate-stimulated hydrogen peroxide (H_2_O_2_) production, and **D** succinate + pyruvate/malate-stimulated H_2_O_2_ production in permeabilized TA muscle bundles. **E** Quantification of mitochondrial fraction of electron leak. **F** Representative immunoblots of gastrocnemius mitochondrial fractions. SOD1 and SOD2 shown as subcellular fraction controls. **G** Quantification of oxidative phosphorylation (OXPHOS) coupling. Quantification of **H** LC3B-II, **I** BNIP3, **J** BNIP3L, **K** PINK1, and **L** PRKN from mitochondrial-enriched fractions. Quantification of **M** CASP9, **N** CASP3, **O** CAPN, and **P** 20S proteasome activity. * p < 0.05 compared to *Atg7*^f/f^ group. *N* = 4–8 mice per group

Autophagy-deficiency completely abolished skeletal muscle mitophagic flux in young P42 *Atg7*^f/f:*Acta1*−Cre^ crossed with mt-Keima reporter mice (i.e., mt-Keima:*Atg7*^f/f:*Acta1*−Cre^; Fig. 5A). Assessment of mitochondrial function in *Atg7*^f/f:*Acta1*−Cre^ mice revealed no changes in maximal respiration, mH_2_O_2_ emission, electron leak fraction, or mitochondrial coupling indicating preserved mitochondrial function (Fig. 5B–E, G). Changes in the mitochondrial localization of autophagy and mitophagy molecules showed a significant reduction in LC3B-II (-90%; Fig. 5F and H) without impacting mitochondrial translocation of BNIP3, PINK1, or PRKN (Fig. 5F, I–L). Ultimately, this demonstrates that tagging of mitochondria by BNIP3, PINK1, and PRKN occurs independent of autophagy.

The absence of phenotypic changes in *Atg7*^f/f:*Acta1*−Cre^ mice despite total ablation of mitophagic flux led us to question whether alternative degradative pathways are compensating for the lack of autophagy. To investigate this, we measured the enzymatic activities associated with various degradation pathways. We found significant elevation of enzymes related to mitochondrial apoptotic signaling, including CASP9 (+ 36%; p < 0.05) and CASP3 (+ 40%; p < 0.05) activity in *Atg7*^f/f:*Acta1*−Cre^ compared to *Atg7*^f/f^ mice (Fig. 5M, N). Additionally, we found elevated CAPN (+ 74%; p < 0.05) and 20S proteasome (+ 87%; p < 0.05) activity in *Atg7*^f/f:*Acta1*−Cre^ compared to *Atg7*^f/f^ mice (Fig. 5O, P). The observed elevation in the activity of enzymes involved in mitochondrial apoptotic signaling, calpain signaling, and the UPS suggests a compensatory mechanism potentially serving as an adaptive response in the absence of autophagy. Collectively, these results point to an adaptive response involving alternative degradative pathways when autophagy is impaired.

**Fig. 6:**
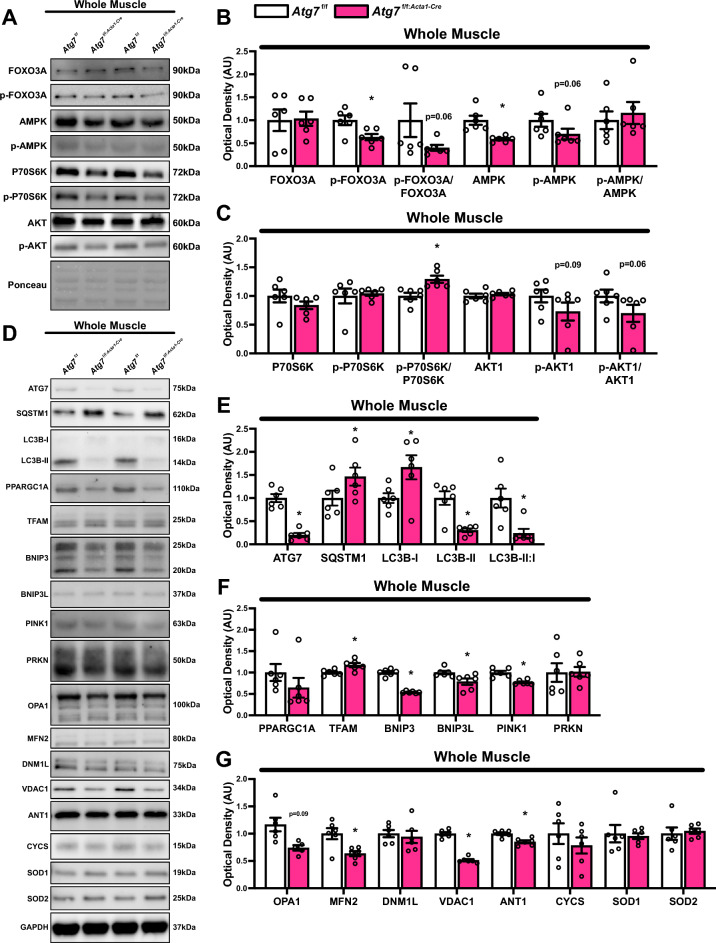
Quantitative analysis of upstream kinases and mitochondrial remodeling proteins in autophagy-deficient skeletal muscle. **A** Representative immunoblots of gastrocnemius lysate of *Atg7*^f/f^ and *Atg7*^f/f:*Acta1*−Cre^ mice. Ponceau stained membrane shown as loading control. **B** Quantitative analysis of FOXO3A, p-FOXO3A (Ser318/321), p-FOXO3A/FOXO3A, AMPK, p-AMPK (Thr172), and p-AMPK/AMPK. **C** Quantification of P70S6K, p-P70S6K (Thr421/424), p-P70S6K/P70S6K, AKT1, p-AKT1 (Thr308), and p-AKT1/AKT1. **D** Representative immunoblots of whole gastrocnemius lysate of *Atg7*^f/f^ and *Atg7*^f/f:*Acta1*−Cre^ mice. GAPDH shown as loading control. **E** Quantitative analysis of ATG7, SQSTM1, LC3B-I, LC3B-II, and LC3B-II:I. **F** Quantitative analysis of PPARGC1A, TFAM, BNIP3, BNIP3L, PINK1, and PRKN. **G** Quantitative analysis of OPA1, MFN2, DNM1L, VDAC1, ANT1, CYCS, SOD1, and SOD2, * p < 0.05 compared to *Atg7*^f/f^. *N* = 6 mice per group

We observed suppression of select catabolic signaling kinases, including a significant reduction in phosphorylated FOXO3A (Ser318/321) and AMPK (p < 0.05), along with trends toward lower p-FOXO3A/FOXO3A ratio and p-AMPK (Thr172) (both p = 0.06; Fig. 6A, B). Additionally, there was a notable increase in the p-P70S6K/P70S6K ratio (Fig. 6A, C), while p-AKT1 (Thr308) and the p-AKT1/AKT1 ratio was reduced, though not statistically significantly (Fig. 6A, C). These findings may suggest aberrant feedback and signaling of kinases that regulate catabolism and anabolism in autophagy-deficient mice. Furthermore, we observed reduced levels of ATG7 and LC3B-II, along with an accumulation of SQSTM1, indicating impaired autophagy in *Atg7*^f/f:*Acta1*−Cre^ mice skeletal muscle (Fig. 6D, E). Investigation into other mitochondrial remodeling processes revealed potential compensatory responses in biogenesis signaling, as shown by elevated TFAM, although PPARGC1A remained unchanged in P42 *Atg7*^f/f:*Acta1*−Cre^ compared to *Atg7*^f/f^mice (Fig. 6D, F). Interestingly, several mitophagy-related molecules including BNIP3, BNIP3L, and PINK1 were significantly suppressed, while PRKN did not change in P42 *Atg7*^f/f:*Acta1*−Cre^ compared to *Atg7*^f/f^ mice (Fig. 6D, F). Regarding mitochondrial dynamics-related proteins, OPA1 and MFN2 were suppressed (Fig. 6D, G), whereas DNM1L did not change (Fig. 6D, G). Additionally, mitochondrial membrane-related molecules VDAC1 and ANT1 were reduced (Fig. 6D, G), with no change in other mitochondria-related proteins including CYCS, SOD1, and SOD2 (Fig. 6D, G).

### MG132 treatment in autophagy-deficient skeletal muscle causes atrophy, weakness, and increased mitochondrial ROS emission

**Fig. 7:**
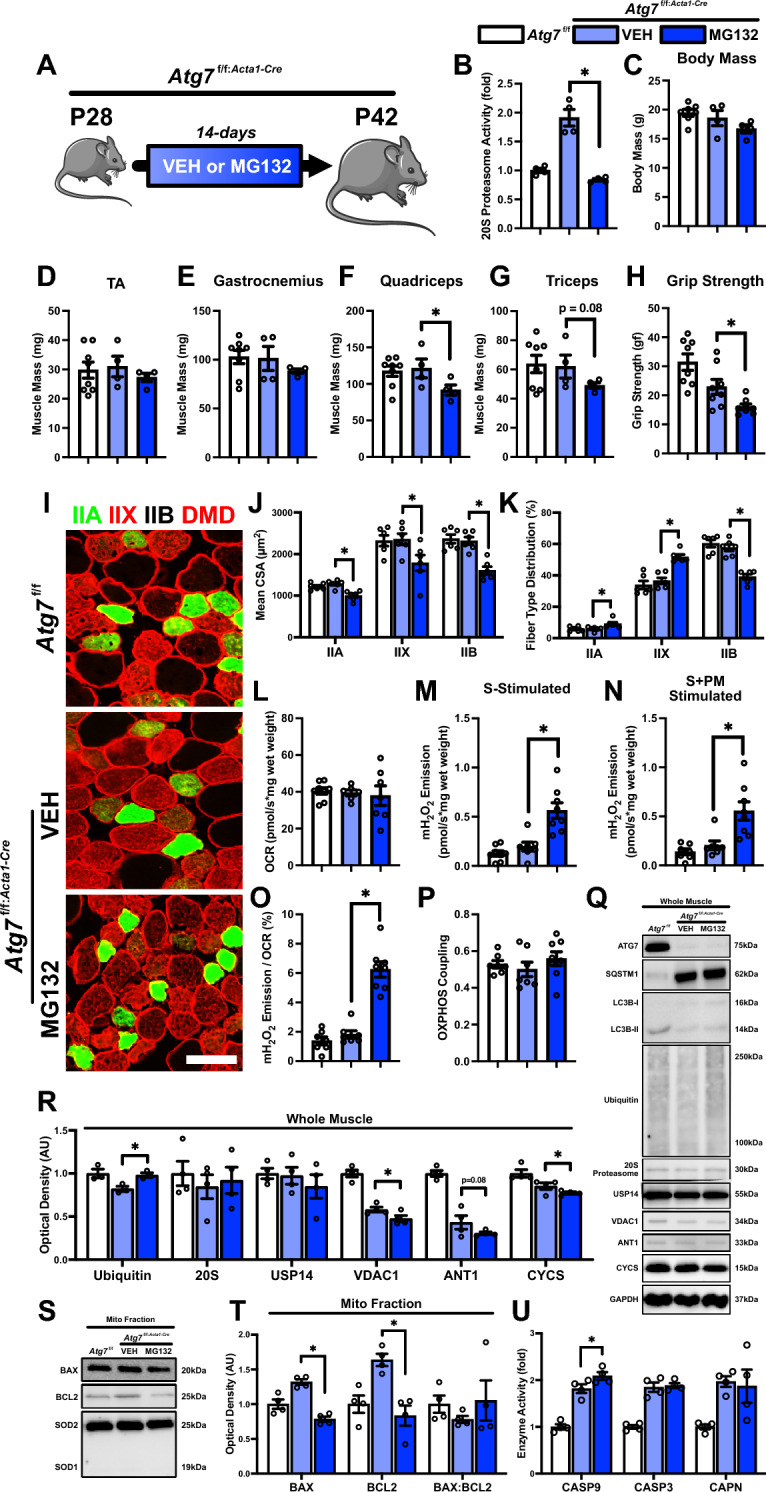
Developmental changes in skeletal muscle of *Atg7*^f/f^ and *Atg7*^f/f:*Acta1*−Cre^ mice treated with either vehicle (VEH) or proteasome inhibitor MG132. **A** Representative visualization of *Atg7*^f/f:*Acta1*−Cre^ mice treated with VEH or MG132 and collected at P42. **B** Quantification of 20S proteasome activity. Quantification of **C** Body mass, skeletal muscle mass of **D** tibialis anterior (TA), **E** gastrocnemius, **F** quadriceps, and **G** triceps, and **H** peak forelimb grip strength. **I** Representative immunofluorescent images of TA muscle cross sections from P42 *Atg7*^f/f^ and *Atg7*^f/f:*Acta1*−Cre^ mice. Samples were stained with antibodies specific for individual MYH isoforms: type IIA (green), type IIX (red), type IIB (unstained), and dystrophin (DMD; red). Scale bars indicate 50 μm. **J**, **K** Quantification of mean cross-sectional area (CSA) and distribution of type IIA, IIX, and IIB fibers. **L** Quantification of maximal oxygen consumption rate (i.e., respiration) of permeabilized bundles from TA muscle. Simultaneous quantification of **M** succinate-stimulated hydrogen peroxide (H_2_O_2_) production, and **N** succinate + pyruvate/malate-stimulated H_2_O_2_ production in permeabilized TA muscle bundles. Quantification of **O** mitochondrial fraction of electron leak and **P** oxidative phosphorylation (OXPHOS) coupling. **Q** Representative immunoblots of whole gastrocnemius lysate of *Atg7*^f/f^ (P42) or *Atg7*^f/f:*Acta1*−Cre^ (P42) mice treated with VEH or MG132. **R** Quantitative analysis of ubiquitin, 20S proteasome, USP14, VDAC1, ANT1, and CYCS. **S** Representative immunoblots of mitochondrial-enriched fraction from the gastrocnemius of *Atg7*^f/f^ (P42) or *Atg7*^f/f:*Acta1*−Cre^ (P42) mice treated with VEH or MG132. **T** Quantitative analysis of mitochondrial localized BAX, BCL2, and BAX:BCL2 ratio. **U** Quantification of CASP9, CASP3, and CAPN activity. * p < 0.05 compared to VEH group. *N* = 4–8 mice per group. *Atg7*^f/f^ group data shown for visualization purposes only

Based on earlier findings that indicated no changes in mitochondrial function, but a reduction in skeletal muscle quality and an upregulation of alternative degradative pathways in response to conditional *Atg7* knockout, we investigated the relevance of these alternative signaling mechanisms. Previous studies have reported a shift towards increased UPS activity in autophagy-deficient muscles, in an attempt to compensate for the diminished autophagy-mediated degradation [16, 17]. Given our observation of elevated UPS activity in autophagy/mitophagy-deficient skeletal muscle, we hypothesized that the UPS could be compensating for the reduced autophagic degradation of cellular components. To test this, we treated *Atg7*^f/f:*Acta1*−Cre^ mice (P28) with either VEH or the proteasome inhibitor MG132 for 14 days, and collected skeletal muscle at P42 (Fig. 7A). MG132 treatment significantly reduced 20S proteasome activity (-55%; p < 0.05; Fig. 7B). Despite no significant changes in body mass, we observed a decrease in quadriceps (-25%; p < 0.05) and triceps (-21%; p = 0.08) mass (Fig. 7C–G). Notably, skeletal muscle quality declined, as evidenced by a reduction in skeletal muscle strength (-30%; p < 0.05; Fig. 7H). This decline was linked to a decrease in CSA of all fiber types (-22% to -30%; p < 0.05; Fig. 7I, J) and a shift toward a slower contractile phenotype (-32% type IIB, + 41% type IIX, + 55% type IIA; p < 0.05; Fig. 7K). Although maximal respiration remained unchanged (Fig. 7L), there was a significant increase (~ threefold) in mH_2_O_2_ emission (succinate stimulated and succinate-pyruvate-malate stimulated) and electron leak (p < 0.05; Fig. 7M–O), indicating impaired mitochondrial function. Immunoblot analysis of whole muscle lysates revealed an accumulation of ubiquitin (+ 20%; p < 0.05; Fig. 7Q, R) without changes in 20S proteasome or USP14 (Fig. 7Q, R). Given the dramatic atrophy and increased mH₂O₂ emission, we next asked whether mitochondrial content was altered under these conditions. We found reductions in VDAC1 (-19%; p < 0.05), ANT1 (-31%; p = 0.08), and CYCS (-10%; p < 0.05) from MG132 treated *Atg7*^f/f:*Acta1*−Cre^ mouse skeletal muscle (Fig. 7Q, R). We then questioned whether these mitochondrial alterations might trigger downstream activation of mitochondrial apoptotic signaling. To explore this, we isolated mitochondrial-enriched fractions from VEH and MG132 treated *Atg7*^f/f:*Acta1*−Cre^ skeletal muscles and performed immunoblot analyses of the pro-apoptotic marker BAX and the anti-apoptotic marker BCL2. Both BAX (-41%; p < 0.05) and BCL2 (-50%; p < 0.05) were reduced in MG132-treated skeletal muscle, but the mitochondrial BAX:BCL2 ratio remained unchanged (Fig. 7S, T). While these findings provide insight into potential changes in mitochondrial apoptotic signaling and mitochondrial outer membrane permeabilization (MOMP), they do not directly confirm BAX and BCL2 interaction. Therefore, we conducted enzymatic activity assays to assess downstream effects of MG132 treatment in *Atg7*^f/f:*Acta1*−Cre^ skeletal muscles. We found a significant increase in CASP9 activity (+ 14%; p < 0.05; Fig. 7U), with no changes in CASP3 or CAPN activity, suggesting that mitochondrial apoptotic signaling is activated when both the UPS and autophagy are impaired.

## Discussion

Skeletal muscle development is a complex, multi-stage process that coordinates cellular responses to enhance muscle mass and functionality. This developmental progression is closely associated with changes in mitochondrial mass, which parallel patterns of skeletal muscle growth [6]. Although several interactions between mitochondrial dynamics and skeletal muscle growth have been identified, the specific impacts of mitochondrial remodeling on skeletal muscle development are not fully elucidated. The present study focused on the role of mitochondrial remodeling during postnatal skeletal muscle development. We report three key findings: (1) significant mitochondrial remodeling occurs concurrently with postnatal skeletal muscle growth, (2) there is a discernible link between skeletal muscle growth, mitochondrial function, and mitophagic flux, and (3) autophagy-deficiency does not adversely affect growth in young mice due to the compensatory activation of the UPS.

### Mitochondrial remodeling defines postnatal muscle development

Postnatal skeletal muscle development involves significant remodeling of contractile components and organelles. Studies have shown increased t-tubule formation [18] and mitochondrial network expansion [7] highlighting dynamic changes during growth. Our RNASeq analyses align with proteomic findings [6], revealing increased expression of genes linked to mitochondrial metabolism and decreased expression of genes for de novo muscle formation. This suggests a shift towards enhancing mitochondrial efficiency to meet the energy demands of growing muscle. Interestingly, *Atg7* expression decreased during skeletal muscle maturation, while *Bnip3* was consistently upregulated throughout this period. Notably, ATG7 protein did not align with transcript levels, as levels peaked at P21, which coincides with elevated mitophagic flux. This was accompanied by increased mitochondrial localization of LC3B-II. Although total BNIP3 levels also increased after P14, mitochondrial localization of BNIP3 remained very low until P42, suggesting a delayed mitochondrial involvement despite the overall increase in protein levels. It remains unclear why this discrepancy occurs and perhaps suggests a cytosolic role of BNIP3.

Our RNA sequencing data is supported by in vivo experiments showing rapid skeletal muscle growth, with body and muscle mass doubling from P21 to P42, consistent with prior studies on muscle mass and fiber size increases [4, 5]. We explored fiber type composition but, unlike earlier reports of MYH IIX and IIB fiber redistribution by P14 [5], found no significant shift from P7 to P42, possibly due to different mouse strains (FVB vs. ICR). We observed enhanced mitochondrial function at P21, marked by increased oxygen consumption and ROS emission. This transient ROS increase may activate hypertrophic signals like MTORC1 [19], supported by the rise in p-P70S6K/P70S6K ratio from P21 onward. Furthermore, the increased mitochondrial ROS emission was accompanied by heightened mitophagic flux, similar to previous findings [20]. Although mitophagy is generally associated with catabolic processes and mitochondrial turnover, its activation does not necessarily suppress anabolic signaling. In fact, transient mitophagy can act as a quality control mechanism that preserves mitochondrial efficiency, thereby sustaining cellular energy production necessary for growth. While excessive ROS levels are often studied for their damaging effects, low to moderate ROS may play a more nuanced regulatory role of finetuning the balance between autophagy and growth pathways [19, 21]. Therefore, the simultaneous activation of mitophagy and MTORC1 may reflect a coordinated response in which ROS acts as a molecular signal that promotes both mitochondrial quality control and anabolic signaling. Closer examination revealed significantly greater mitochondrial localization of BNIP3, PINK1, and PRKN. Notably, mitochondrial localized BNIP3 exhibited more than a ten-fold increase during development, aligning with both our mitophagy observations and *Bnip3* RNA sequencing data. Elevated levels of these mitophagy receptor molecules have been previously documented [6]. To our knowledge, this study is the first to explore potential molecules targeting mitochondria during development, providing new insights into the molecular mechanisms of skeletal muscle growth and adaptation. We postulate that the transient elevation in ROS observed at P21 may function as a molecular trigger for the activation of hypertrophic kinases and the induction of mitochondrial remodeling processes such as mitophagy. This cascade of events is proposed to facilitate the development of more efficient mitochondria, thereby meeting the increased energetic demands of growing fibers.

Prior studies have highlighted significant remodeling of the mitochondrial network during skeletal muscle development, transitioning from a “parallel” to a “perpendicular” orientation [6]. This reorganization is believed to support the maturation of the sarcoplasmic reticulum (SR), thereby regulating NADH flux and mitochondrial membrane potential, ultimately enhancing mitochondrial functionality [6, 18, 22, 23]. In our current investigation, we utilized mt-Keima mice to examine mitochondrial morphology, and observed that the mitochondrial network maintained a “parallel” morphology, showing no significant morphological changes. Immunoblot analysis of mitochondrial dynamics proteins revealed stable levels of fusion proteins OPA1 and MFN2, but a noticeable reduction in the fission protein DNM1L at P21 and P42. Genetic differences between our FVB reporter mouse model and the previously studied C57BL/6N strain could be a potential factor contributing to the observed differences in our results [6], or perhaps limitations in our imaging resolution that preclude detection of subtle or three-dimensional structural changes [6, 24].

### Link between skeletal muscle growth, mitochondrial function, and mitophagy during development

Skeletal muscle research has traditionally focused on growth, but recent studies highlight the importance of degradative mechanisms like autophagy and mitophagy [8, 25–27]. These processes, activated by cellular stress, remove damaged materials, including mitochondria [8, 25–27]. Their role in regulating healthy development is gaining attention. Though studies on these pathways during muscle development are limited, evidence shows that inhibiting autophagy or mitophagy impairs muscle formation in vitro [13–15]. One proposed mechanism suggests that deficiencies in autophagy and mitophagy disrupt signaling pathways essential for mitochondrial biogenesis [13, 14]. Data from the present study highlight a significant increase in mitophagic flux concurrent with a reduction in mitochondrial biogenesis signaling, alongside stable levels of mitochondrial-related proteins in developing skeletal muscle tissue. These findings suggest that during myogenesis, as opposed to postnatal skeletal muscle development, mitochondrial quality control might be predominantly governed through enhanced degradation rather than biogenesis. This shift towards prioritizing mitochondrial integrity via increased mitophagic flux, without alterations in other mitochondrial remodeling processes, may offer insights into the complex interplay of cellular mechanisms driving muscle development.

In the present study, we observed a notable increase in mitochondrial ROS emission at P21, which coincided with enhanced mitochondrial localization of autophagy-related proteins LC3B-II, PINK1, and PRKN. Simultaneously, the mitophagy receptor BNIP3 exhibited a striking 14-fold increase by P42, whereas BNIP3L rapidly declined after P7 to undetectable levels. This opposing expression pattern may reflect differences in transcriptional regulation, functional compensation, or developmental specificity of BNIP3 as the primary mitophagy receptor. Nonetheless, the onset of increased mitophagic flux beginning at P21 suggests a tightly regulated temporal control of these processes. The role of mitochondrial ROS in triggering mitophagy is well-documented [28], and our findings promote suggest that the surge in ROS levels may promote PINK1/PRKN-mediated mitophagy during early developmental stages. Subsequently, BNIP3-mediated mitophagy may become more prominent as the muscle continues to mature, considering that it is only elevated at P42.

### Autophagy-deficiency impairs skeletal muscle strength and promotes UPS and mitochondrial apoptotic signaling without affecting overall growth

The first studies using muscle-specific autophagy-deficient models revealed significant growth defects in young autophagy-deficient mice. For example, previous reports show that animals with muscle-specific autophagy-deficiency exhibited a reduction in body mass from P40 and the disparity continues as the animals age [17]. Furthermore, these animals demonstrated a greater loss in muscle size and function after five months of autophagy-deficiency [17]. Our laboratory has built on these findings, showing that defects in autophagy-deficient muscle manifest over time. Specifically, we observed skeletal muscle weakness in the soleus at 15 weeks, which was not evident at 5 weeks [12]. Additionally, we found a significant increase in centralized nuclei in the extensor digitorum longus (EDL) at 15 weeks compared to 5 weeks [12]. These observations highlight a divergent age-specific and fiber type/muscle-specific response in autophagy-deficient muscles [12]. Data from the present study are consistent with our previous work showing minimal changes in body mass, skeletal muscle mass, or fiber type distribution in 5 week old *Atg7*^f/f:*Acta1*−Cre^ mice, yet we noted skeletal muscle weakness in these animals [12]. This phenomenon has been previously observed, with studies demonstrating that although muscle mass is maintained in aged TRIM63 knockout models, these animals exhibit a reduction in peak muscle force [29]. The reduction in muscle quality found in the present study may be a result of muscle damage or impairments in contractile ability in the *Atg7*^f/f:*Acta1*−Cre^ mice [12]. Further supporting the importance of autophagy in skeletal muscle integrity, a recent study identified MYTHO as a key regulator of skeletal muscle homeostasis. Chronic MYTHO knockdown led to progressive and pathological skeletal muscle hypertrophy, driven by sustained activation of MTORC1 signaling [30]. In line with this, we observed a compensatory increase in the p-P70S6K/P70S6K ratio in *Atg7*^f/f:*Acta1*−Cre^ mice, suggesting enhanced MTORC1 signaling in response to impaired autophagy. Despite these findings, discrepancies remain between studies. While our data highlight selective functional deficits without overt atrophy in early stages, other groups have reported significant reductions in both skeletal muscle size and function in autophagy-deficient models [17]. These differences may reflect variation in model design, age of assessment, or fiber-type sensitivity to autophagy loss.

Significant remodeling of the mitochondrial network occurs during postnatal skeletal muscle development, as documented in various studies [6, 7, 24]. Previous research has established that constitutive autophagy-deficiency in skeletal muscle leads to the accumulation of abnormal/swollen and dysfunctional mitochondria at 8-weeks of age [17]. Our confocal microscopy analyses using autophagy-deficient mt-Keima mice did not reveal any significant changes in mitochondrial network morphology. Additionally, we observed no changes in mitochondrial function, such as maximal respiration or ROS emission, despite complete ablation of mitophagic flux. This finding contrasts with other studies reporting significant mitochondrial dysfunction in autophagy-deficient states [17]. The apparent absence of defects in our young animal models may be attributed to compensatory degradative mechanisms. For instance, numerous studies have demonstrated an upregulation of apoptotic signaling, ER stress signaling, and UPS in an autophagy-deficient state [12, 13, 15, 16, 31–33]. Although the UPS can compensate for impaired autophagy-mediated protein degradation, its role is limited to degrading short-lived, soluble proteins and may be transient [34]. The formation of large, insoluble protein aggregates in autophagy-deficient states can lead to cellular dysfunction, potentially explaining the age-related exacerbation of defects observed in our studies [12]. In addition to these, there is evidence suggesting that alternative lysosomal degradation pathways, such as chaperone-mediated autophagy (CMA) and microautophagy, may contribute to maintaining proteostasis and mitochondrial quality in the absence of canonical autophagy. The selective degradation of proteins containing a KFERQ-like motif through CMA has been shown to influence mitochondrial function indirectly through the regulation of mitophagy molcules like PRKN [35]. Alternatively, the direct invagination of the lysosomal membrane to sequester cytosolic material via microautophagy may also play a limited but compensatory role in the turnover of small damaged mitochondrial components [36]. While these alternative pathways are unlikely to fully compensate for the loss of macroautophagy, their activation may help delay the onset of mitochondrial and skeletal muscle dysfunction during the early stages of autophagy deficiency. However, further research is needed to clarify the extent of their contribution in this context.

### UPS activity compensates for lack of autophagy signaling to protect skeletal muscle from mitochondrial dysfunction and atrophy

There is significant interplay between degradative pathways in skeletal muscle. Evidence suggests that when autophagy signaling is impaired, there is compensatory activation of the UPS, and vice versa [16, 31–33]. In the present study, we observed similar findings: autophagy deficiency in skeletal muscle led to compensatory activation of the UPS and mitochondrial apoptotic signaling in young mice. As a result, we questioned whether the compensatory activation of the UPS was protecting against functional defects in autophagy-deficient animals. Upon treating *Atg7*^f/f:*Acta1*−Cre^ mice with MG132, we observed significant dysfunction. Notably, we found a significant reduction in skeletal muscle quality (i.e., grip strength) and greater atrophy in autophagy-deficient mice treated with MG132. This is likely due to the accumulation of cellular debris, as both autophagy and the UPS are critical for maintaining proteostasis in skeletal muscle. Additionally, these findings may reflect underlying neuromuscular dysfunction, as the intraperitoneal injections of MG132 could affect not only skeletal muscle but also other tissues, including neuronal proliferation [37], potentially impairing neuromuscular junction integrity and maturation during the developmental period. Interestingly, we observed a marked reduction in type IIB fibers, with a shift towards type IIA and IIX fibers. This shift may be attributed to fast/glycolytic muscles being more sensitive to autophagy deficiency and having higher autophagic flux [12, 38]. In support of this, previous work from our group has shown that autophagy inhibition during immobilization leads to a selective loss of type IIB fibers, accompanied by a relative increase in type IIX and IIA fibers [39]. These findings suggest that highly glycolytic fibers are more susceptible to the absence of autophagy, which may ultimately compromise muscle fiber integrity. Additionally, MG132 treatment in autophagy-deficient mice led to the activation of CASP9, without changes in CASP3 or CAPN activity. The activation of CASP9 was also associated with increased mitochondrial ROS emission, potentially due to CASP9-mediated cleavage and activation of other pro-apoptotic molecules such as BID, which can localize to mitochondria and enhance ROS production. Taken together, the absence of short-term detrimental effects from autophagy deficiency may be explained by the compensatory activation of the UPS, which may provide temporary protection against muscle dysfunction. However, the long-term consequences of these disruptions in proteostasis remain to be fully elucidated.

## Conclusion

In conclusion, our findings underscore the pivotal role of mitochondrial remodeling in postnatal skeletal muscle development. The significant remodeling observed during this stage indicates that mitochondrial adaptations are closely linked to skeletal muscle growth patterns. Our study highlights that while autophagy-deficiency can impair skeletal muscle strength and mitophagy, it does not adversely affect overall skeletal muscle growth in mice due to the compensatory activation of the UPS. These insights contribute to a better understanding of the intricate processes underlying muscle development and suggest that targeting mitochondrial pathways could offer novel therapeutic strategies for muscle-related diseases. Future research should focus on exploring these pathways in greater detail to uncover potential interventions for enhancing muscle function.

## Material and methods

### RNA sequencing analysis

RNA sequencing analyses were performed in a similar manner as previously described [39]. Publicly available RNA sequencing datasets were retrieved from the Gene Expression Omnibus (GEO) database (accession number GSE1025). Initial quality control was based on exclusion criteria including adapter contamination and low-quality bases. Gene-level expression was quantified and analyzed using the DESeq2 package in RStudio. Differential expression was determined with an adjusted p-value of < 0.05 and a minimum log2 fold change of > 1, identifying significantly differentially expressed genes. Enrichment of biological processes and pathways was assessed using the clusterProfiler package, with tests against Gene Ontology (GO) terms. Pathways were considered significantly enriched at a corrected p-value of < 0.05. Visualization of the results, including volcano plots, heatmaps, and barplots, was conducted using GraphPad PRISM software.

### Animals

Mt-Keima mice with a FVB background were kindly gifted by Torin Finkel (University of Pittsburgh) [40, 41]. These animals are a valuable tool for studying mitophagy since they express the fluorescent Keima protein conjugated to the COX8 subunit found within complex IV in the IMM. The unique feature of the Keima molecule is its dual-excitation, single-emission fluorescence, which changes depending on the pH of its environment. Under neutral pH conditions, as found in the cytosol, Keima is excited at 488 nm. In the acidic conditions of the lysosome, where mitochondria are degraded, the excitation shifts to 561 nm. This shift allows for precise monitoring of mitophagy. Pure-bred mt-Keima pups were examined at several postnatal timepoints (P7, P14, P21, and P42) during the initial characterization. Weaning took place at P21, and as a result it was difficult to determine biological sex using anogenital distance at the earlier timepoints. All pups were randomly selected at P7 and P14 timepoints. Equal number of male and female animals were collected at P21 and P42.

C57BL/6 homozygous for the floxed *Atg7* allele (*Atg7*^f/f^; kindly provided by Dr. Herbert Virgin, Washington University) were crossed with carriers of the constitutive skeletal muscle-specific Cre recombinase under the control of the actin alpha 1 promotor (i.e., *Acta1*-Cre-; kindly provided by Dr. Karyn Esser, University of Florida). Breeding of these animals ultimately generated a constitutive skeletal muscle-specific *Atg7* knockout model (*Atg7*^f/f:*Acta1*−Cre^). *Atg7*^f/f^ littermates were used as controls. Given the low ACTA1 levels and lack of early developmental defects observed in previous work until P40 [17], we decided to collect these animals at P42 to understand the effect of *Atg7*-deficiency on young skeletal muscle. Additional experiments were performed by breeding mt-Keima mice with *Atg7*^f/f^ animals to generate mt-Keima:*Atg7*^f/f^ mice, which were then crossed with *Acta1*-Cre mice to ultimately generate mt-Keima:*Atg7*^f/f:*Acta1*−Cre^ mice. These mice were utilized to confirm loss of mitophagic flux. Further experiments were conducted where vehicle (VEH; 5% DMSO, 40% PEG300 and 5% Tween-20 in saline) or MG132 (10 mg/kg/day dissolved in VEH) were injected daily from P28 to P42 to inhibit proteasome activity.

Animals were given free access to food and water. All animal procedures were conducted in accordance with the standards set by the Canadian Council on Animal Care (CCAC) and were approved by the Animal Care Committee (ACC) at the University of Waterloo.

### Isolation of skeletal muscle

The tibialis anterior (TA), gastrocnemius, quadriceps, and triceps brachii muscles were rapidly excised. One TA was stored in BIOPS buffer (2.77 mM CaK_2_EGTA, 7.23 mM K_2_EGTA, 5.77 mM Na_2_ATP, 6.56 mM MgCl_2_-6H_2_O, 20 mM taurine, 15 mM Na_2_PCr, 20 mM imidazole, 0.5 mM DTT, 50 mM K-MES) on ice for respirometry and live microscopy imaging. The contralateral TA was embedded in OCT and frozen in isopentane cooled by liquid nitrogen for MYH analysis. One gastrocnemius from each animal was snap frozen in liquid nitrogen for whole tissue immunoblot analyses, while the other was stored in PBS-EDTA solution (10 mM EDTA in 1X PBS, pH 7.4) for mitochondrial fractionation. Quadriceps and triceps were only used for mass measurements.

### Live fiber imaging and mitophagy flux analyses

Live fiber imaging and analyses were performed as previously described [39]. The TA muscle was manually separated into bundles in BIOPS buffer on ice. Bundles were rapidly transferred to #1 coverslips and carefully separated to allow maximal contact of muscle bundle to the coverslip. A thin layer of 0.8% agarose gel was placed on top of the bundles to prevent movement during imaging. Five to ten fields consisting of 3–5 fibers were imaged on a Zeiss LSM 800 confocal microscope at 40X magnification. Individual excitation wavelengths were used to minimize overlap between fluorescent channels. An excitation wavelength of 488 nm was used to determine healthy mitochondria (pH ~ 7; green), whereas an excitation wavelength of 561 nm was used to determine mitochondria undergoing mitophagy (pH ~ 4; red). Emission wavelengths remained the same at 620 nm. Puncta count and size were determined in each field using the automated Particle Analysis function in ImageJ.

### High-resolution respirometry with simultaneous ROS measurements

Respirometry experiments were performed as previously described [39]. The TA muscle was separated under a stereomicroscope into bundles in ice-cold BIOPS buffer. Bundles were permeabilized in 50 µg/ml of saponin in 1.5 ml BIOPS buffer for 30 min on ice with gentle rocking. Muscle bundles (1–3 mg) were then transferred to 1.5 ml of buffer Z (105 mM K-MES, 30 mM KCl, 1 mM EGTA, 10 mM K_2_HPO_4_, 5 mM MgCl_2_-6H_2_O, 5 mg/ml BSA, pH 7.1) to wash prior to respirometry experiments. Oxygen consumption and mitochondrial hydrogen peroxide (mH_2_O_2_) emission was measured in buffer Z supplemented with 10 mM blebbistatin, 10 mM Amplex Red, 5U/ml horseradish peroxidase, and 10U/ml superoxide dismutase using an Oxygraph-2K equipped with a LED2 fluorometry module. Experiments were conducted at 37 °C in a 2 ml chamber that was hyperoxygenated to ~ 350 uM. Reverse electron flow was determined with the addition of 10 mM succinate, followed by 5 mM pyruvate and 2.5 mM malate. Maximal coupled respiration was stimulated with the addition of 5 mM ADP. Cytochrome *c* was added to ensure outer membrane integrity. mH_2_O_2_ emission was calibrated from a standard curve established with the same reaction conditions.

### Mitochondrial fractionation

Mitochondrial fractions were isolated similar to previously described [13, 39, 42–44]. Gastrocnemius was chopped for one minute and transferred back into a tube containing 5 ml of PBS with 10 mM EDTA (PBS-EDTA solution) and centrifuged at 500 g for 5 min. The supernatant was discarded, and muscle digested in 0.05% trypsin in PBS-EDTA solution for 5 min on ice. The trypsin solution was removed by centrifugation at 500 g for 5 min. Digested muscle was resuspended in mitochondrial isolation buffer (100 mM KCl, 50 mM MOPS, 5 mM MgSO_4_-7H_2_O, 1 mM EGTA, 2 mg/ml BSA, pH 7.4) and homogenized using a Teflon homogenizer equipped on a drill press set to 200 rpm for 5–10 strokes. The homogenized tissue was subjected to low-speed centrifugation (~ 700 × *g*) to pellet large debris and the supernatant was transferred to ultracentrifuge tubes and spun at 10,000 g for 15 min at 4 °C. The resulting pellet constituted the mitochondrial pellet and was resuspended in mitochondrial isolation buffer without BSA before being transferred to 1.5 ml microcentrifuge tubes and spun at 10,000 g for 5 min. The resulting pellet was washed and centrifuged three times in mitochondrial isolation buffer without BSA to remove any cytosolic protein. After the final spin, the supernatant was aspirated, and the pellets were frozen in liquid nitrogen.

### Immunoblotting

Immunoblot analyses were performed as previously described [39, 43, 45]. Snap frozen muscle was divided into 20–30 mg portions and stored in separate tubes. Muscles were crushed into a powder using a liquid nitrogen-cooled pestle and mortar, and transferred to 1.5 ml tubes containing 5 volumes of lysis buffer (20 mM HEPES, 10 mM NaCl, 1.5 mM MgCl_2_, 1 mM DTT, 20% glycerol, 0.1% Triton-X100, pH 7.4) with protease and phosphotase inhibitors. Mitochondrial enriched fractions were resuspended in lysis buffer with protease inhibitors and sonicated at 40 Hz for 10 s on ice to dissolve any membranal structures. Protein content was determined using a BCA protein assay. Equal protein was loaded and separated using 8–14% SDS-PAGE, transferred on to PVDF membranes, and blocked with 2–5% non-fat milk powder in TBS-T at room temperature for 1 h. Membranes were briefly rinsed and incubated overnight in primary antibodies against: BNIP3 (#3769, Cell Signaling), BNIP3L (#12396, Cell Signaling), ATG7 (#8558, Cell Signaling), MAP1LC3B/LC3B (#2775, Cell Signaling), GAPDH (#2118, Cell Signaling), OPA1 (#80471, Cell Signaling), MFN2 (#9482, Cell Signaling), DNM1L (#8570, Cell Signaling), FOXO3A (#9467, Cell Signaling), p-FOXO3A (#9465, Cell Signaling), AMPK (#2532, Cell Signaling), p-AMPK (#2535, Cell Signaling), P70S6K (#2708, Cell Signaling), p-P70S6K (#9204, Cell Signaling), AKT1 (#9272, Cell Signaling), p-AKT1 (#9275, Cell Signaling), SQSTM1 (PM045, MBL), PINK1 (sc-33796, Santa Cruz), PRKN (sc-32282, Santa Cruz), VDAC1 (sc-390996, Santa Cruz), ANT1 (sc-9299, Santa Cruz), CYCS (sc-13256, Santa Cruz), TFAM (sc-166965, Santa Cruz), PPARGC1A (ST1202, Sigma), SOD1 (SOD-101, Stressgen), SOD2 (SOD-110, Stressgen), 4HNE (ab46545, Abcam), ubiquitin (sc-8017, Santa Cruz), 20S proteasome (sc-166073, Santa Cruz), and USP14 (sc-398009, Santa Cruz). Following incubation, membranes were washed, and appropriate horseradish peroxidase-conjugated secondary antibody was applied at room temperature for 1 h. Bands were visualized using ECL substrate on a ChemiDoc Imaging System. Densitometric analysis was performed using ImageLab software (Bio-Rad), and results are presented as normalized optical density values.

### Caspase, calpain, and proteasomal activity assay

To measure caspase (CASP), calpain (CAPN), and proteasomal activity, whole‐muscle lysates were collected as above in the absence of protease inhibitors [39, 42, 46, 47]. Samples were incubated in duplicate with 20 μM of Ac‐DEVD‐AFC (AAT Bioquest, 13,401) for CASP3 or Ac‐LEHD‐AFC (Tocris Bioscience, 1575) for CASP9 in assay buffer (20 mM HEPES pH 7.4, 10 mM DTT, and 10% glycerol). For CAPN activity, samples were incubated in duplicate with 20 μM of Suc-LLVY-AMC (Enzo Life Sciences, BML-P802) and Z-LL-CHO (Enzo Life Sciences, BML-PI116) inhibitor in assay buffer for 2 h at 30 °C. For 20S proteasomal activity, samples were incubated in duplicate with 20 μM of Suc-LLVY-AMC and MG132 (Adooq Bioscience, A11043) inhibitor in proteasome assay buffer (50 mM Tris/HCl, 25 mM KCl, 10 mM NaCl, 1 mM MgCl_2_; pH 7.5) for 1 h at 30 °C. Fluorescence measurements were performed at room temperature using a Cytation 5 Imaging Multi‐Mode Reader. The AFC probe was measured with excitation and emission wavelengths at 400 and 505 nm, respectively. The AMC probe was measured with excitation and emission wavelengths at 360 and 440 nm, respectively. The fluorescence values were normalized to protein concentration of samples and expressed as fold changes in fluorescence.

### Immunofluorescence fiber type analysis

TA muscle was embedded in OCT compound (Tissue Tek), frozen in isopentane cooled by liquid nitrogen, and stored at −80 °C until analysis. Ten-micron cryosections were cut, mounted onto microscope slides and maintained at −20 °C until staining, as previously described [12, 39, 48]. For staining, slides were dried at room temperature, circumscribed with a hydrophobic pen and blocked using 10% goat serum in PBS. Slides were incubated at room temperature with antibodies against type IIA (SC-71, DSHB), type IIX (6H1, DSHB), and dystrophin (MANDYS1, DSHB) for 2 h. Slides were washed three times with PBS and incubated at room temperature with appropriate secondary antibodies. Following incubation with secondary antibodies, slides were washed three times and mounted with Fluoromount-G (Thermo Fisher Scientific) mounting medium. Fiber cross-sectional area (CSA) and fiber counts was quantified using ImageJ.

### Grip strength measurement

Grip strength was assessed as an indicator of skeletal muscle strength using a custom-built grip apparatus. The grip apparatus featured a custom 3D-printed design using PLA filament and incorporated a micro load cell connected to a HX711 amplifier, all managed by an Arduino Uno microcontroller. The microcontroller was programmed using the Arduino IDE to record peak grip efforts at 3-s intervals. Data were saved to a spreadsheet for subsequent analysis. Maximal forelimb grip strength was determined from 3–5 trials per mouse. Data are presented in gram-force (*g*f).

### Statistics

Statistical analyses were performed in GraphPad PRISM. T-tests were performed to compare developmental changes from all groups compared to P7. T-tests were also performed to compare *Atg7*^f/f^ to *Atg7*^f/f:*Acta1*−Cre^ or VEH to MG132 treatment groups. An alpha value of 0.05 was considered statistically significant.

## Data Availability

The datasets during and/or analysed during the current study are available from the corresponding author on reasonable request.

## Abbreviations

4HNE: 4-Hydroxynonenal
AKT1: AKT serine/threonine kinase 1
AMPK: AMP-activated protein kinase
ANT1: Adenosine nucleotide translocator 1
ATG7: Autophagy related 7
BNIP3: BCL2 interacting protein 3
BNIP3L: BNIP3 like
CAPN: Calpain
CASP: Caspase
CYCS: Cytochrome c
DNM1L: Dynamin 1 like
FOXO3A: Forkhead box O3 alpha
MAP1LC3B/LC3B: Microtubule associated protein 1 light chain 3 beta
MFN2: Mitofusin 2
OPA1: Optic atrophy 1
P70S6K: P70 ribosomal S6 kinase
PINK1: PTEN induced kinase 1
PPARGC1A: PPARG coactivating 1 alpha
PRKN: Parkin
SOD: Superoxidase dismutase
SQSTM1: Sequestosome 1
TA: Tibialis anterior
UPS: Ubiquitin–proteasome system
USP14: Ubiquitin specific peptidase 14
VDAC1: Voltage dependent anion channel 1
